# Toward Rapid Detection of Viable Bacteria in Whole Blood for Early Sepsis Diagnostics and Susceptibility Testing

**DOI:** 10.1021/acssensors.1c01219

**Published:** 2021-08-19

**Authors:** Sharath Narayana Iyengar, Jiri Dietvorst, Amparo Ferrer-Vilanova, Gonzalo Guirado, Xavier Muñoz-Berbel, Aman Russom

**Affiliations:** † Division of Nanobiotechnology, Department of Protein Science, Science for Life Laboratory, KTH Royal Institute of Technology, Stockholm 17165, Sweden; ‡ AIMES - Center for the Advancement of Integrated Medical and Engineering Sciences at Karolinska Institutet and KTH Royal Institute of Technology, Stockholm 17165, Sweden; § Instituto de Microelectrónica de Barcelona (IMB-CNM, CSIC), Universitat Autónoma de Barcelona, Cerdanyola del vallès, Barcelona 08193, Spain; ∥ Department de Química, Universitat Autònoma de Barcelona, Cerdanyola del Vallès, Barcelona 08193, Spain

**Keywords:** sepsis, bacteria, *E. coli*, selective cell lysis, Prussian blue, colorimetric, blood

## Abstract

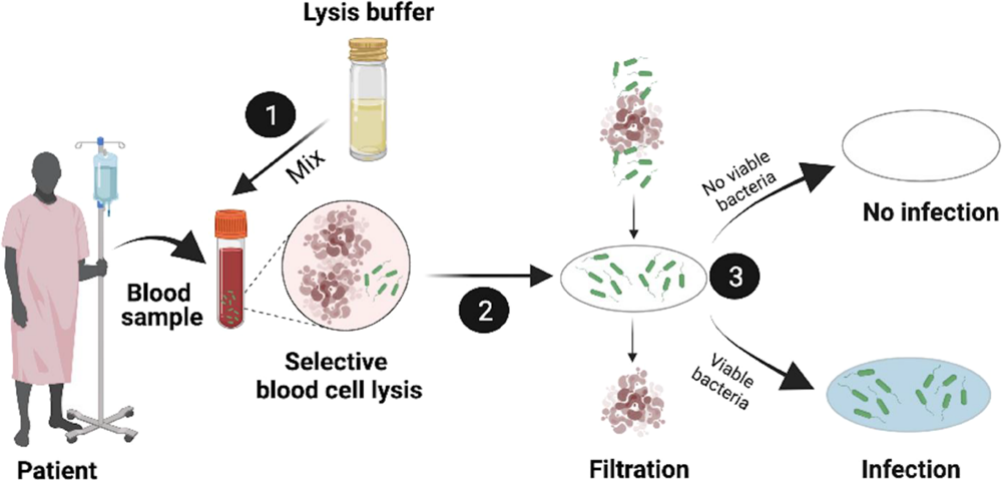

Sepsis is a serious bloodstream infection where the immunity of the host body is compromised, leading to organ failure and death of the patient. In early sepsis, the concentration of bacteria is very low and the time of diagnosis is very critical since mortality increases exponentially with every hour after infection. Common culture-based methods fail in fast bacteria determination, while recent rapid diagnostic methods are expensive and prone to false positives. In this work, we present a sepsis kit for fast detection of bacteria in whole blood, here achieved by combining selective cell lysis and a sensitive colorimetric approach detecting as low as 10^3^ CFU/mL bacteria in less than 5 h. Homemade selective cell lysis buffer (combination of saponin and sodium cholate) allows fast processing of whole blood in 5 min while maintaining bacteria alive (100% viability). After filtration, retained bacteria on filter paper are incubated under constant illumination with the electrochromic precursors, i.e., ferricyanide and ferric ammonium citrate. Viable bacteria metabolically reduce iron(III) complexes, initiating a photocatalytic cascade toward Prussian blue formation. As a proof of concept, we combine this method with antibiotic susceptibility testing to determine the minimum inhibitory concentration (MIC) using two antibiotics (ampicillin and gentamicin). Although this kit is used to demonstrate its applicability to sepsis, this approach is expected to impact other key sectors such as hygiene evaluation, microbial contaminated food/beverage, or UTI, among others.

Sepsis is a serious medical condition characterized by a whole-body inflammatory state due to bloodstream infection, with *Escherichia coli* (*E. coli*) and *Staphylococcus* sp. (*Staph*) as the most leading causes of infection.^1^ In sepsis, bacteria reach the bloodstream from a local region of infection and spread, leading to organ dysfunction and, in the most severe conditions, to the patient’s death.^2^ According to the 2018 statistics from the World Health Organization (WHO), about 30 million people are affected by sepsis worldwide, including 3 million newborn kids and 1.2 million children. Regarding mortality, 6 million people die of sepsis in the world every year, 500 thousand of them are newborn kids, and this condition is also responsible for 1 in 10 maternal deaths. This high mortality is mostly associated with the difficulty of diagnosing sepsis at its early stages: initial bacterial concentration does not exceed 100 colony forming units (CFU)/mL^3^ and every hour of delay in the diagnostic and treatment increases the mortality of patients up to 10%.^4^ These fast diagnostic requirements are not attainable with traditional golden standards, i.e., cell culture and polymerase chain reaction (PCR) amplification methods, which require between 24 and 72 h before appropriate antibiotics could be prescribed to the patients.^5−7^

Better performances are obtained with sepsis diagnosis kits currently available in the market, e.g., IRIDICA, SeptiFast, SeptiTest, or U-them. These kits combine lysis buffers, for fast blood sample pretreatment and DNA extraction, with PCR analysis, enabling the detection of bacterial DNA, and thus diagnosing sepsis within 4 and 8 h. The main limitations of these kits are their low sensitivity and specificity due to the background from the human DNA and the impossibility to distinguish between DNA coming from live or dead bacteria.^8^ Moreover, since bacteria are lysed to extract DNA, it is not possible to perform antimicrobial susceptibility tests and medical prescriptions are based on genetic information, e.g., the presence of resistance genes. Although some technologies for viable bacteria isolation are now being developed based on microfiltration, they suffer from blood clogging and low throughput.^9^ On the other hand, many strategies based on electrochromic metabolic indicators are available commercially or being developed to distinguish between live and dead bacteria.^10^ These bioassays are based on the use of a redox molecule that is reduced due to bacterial metabolism changing its color or resulting in the production of a fluorescent compound, e.g., Presto Blue or Alamar Blue. As a result, the presence of live bacteria can be detected after 10–15 h of incubation.

Alternative promising technologies are now being developed based on different detection strategies. Chu *et al*.,^11^ developed a colorimetric sensor for the detection of low concentrations (5–300 CFU/mL) of eight strains of bacteria using tryptic soy broth (TSB) media. Although high sensitivity is demonstrated, this method has two important drawbacks, namely, (i) the need for sample pretreatment when using real blood samples (ii) and the duration of the assay, which expands up to 24 h.^11^ This long incubation time is the most limiting aspect of these bioassays for their implementation as routine techniques. Other approaches are based on the detection of biomarkers in blood for early sepsis diagnosis. Rios-Toro *et al*.,^12^ Buchegger and Preininger,^13^ and Kemmler *et al.*(14) demonstrated that interleukin-6 (IL-6), C-reactive protein (CRP), and procalcitonin (PCT) may be good candidates for diagnosing severe sepsis and septic shock cases, the latter developing a point-of-care (POC) device for sepsis detection. Min *et al.*, on the other hand, demonstrated that another potential biomarker, IL-3, could be detected with high sensitivity and specificity using a magneto-electrochemical sensor.^15^ Ghonge *et al.*(16) and Hassan *et al.*(17) measured CD-64 biomarkers in whole blood using a microfluidic biochip with integrated smartphone imaging in 50 min, and Zhang *et al.*(18) and Zhou *et al.*(19) also developed a microfluidic chip to capture CD-64 and CD-69 and to detect them within 2 h, providing results that are significantly different between healthy and sepsis patient samples. Klouche *et al.* measured the levels of the CD-14 subtype (presepsin) as a biomarker to diagnose community-acquired pneumonia in 44 ICU patients.^20^ These studies are highly interesting but rely on the specificity of these biomarkers for sepsis. Phua *et al.*, for example, reported that patients showing systemic inflammatory response syndrome had negative culture tests, raising the question of the true influence of infection for the observed inflammatory response.^22,23^ Other mimickers of sepsis can be due to tissue injury, thyroid storm, or inflammatory disorder, among others.^23−25^ In addition, the increased levels of IL-6, PCT, and CRP biomarkers have also been reported in the early and later stages of the current viral pandemic COVID-19 and in patients with hypoxemia.^26,27^ In addition, high to moderate levels of these biomarkers in the blood are needed for effective detection,^28^ which indicates the need for patient samples who are in the later stage of sepsis. It has also been found that one-third of the cases in ICU patients with pneumonia had viral infections found by PCR assays.^23,29^ Thus, this approach makes it impossible to differentiate between a viral and bacterial infection and does not provide any information on antibiotic susceptibility, where the clinicians have to still depend on blood cultures to prescribe a specific antibiotic. Thus, there is an urgent need for efficient methods to diagnose sepsis in a very short time, here attained by selective isolation of viable bacteria from whole blood and their rapid detection with a highly sensitive photocatalytic colorimetric approach. This kit for sepsis diagnosis combines two reagents. On the one hand, the composition of the lysis buffer is adjusted to ensure fast and efficient rupture of blood mammalian cell membranes while preserving the bacterial cells’ integrity and viability. On the other hand, a metabolic indicator was implemented for sensitive live bacteria detection, which also allowed fast antibiotic susceptibility testing. Bacteria detection is achieved through a photocatalytic approach based on a cyanotype reaction where the metabolic production of Prussian blue (PB) is used to detect the presence of live bacteria.^21^ The starting iron donors for PB formation are a specific concentration mixture of potassium ferricyanide and ferric ammonium citrate. Bacteria readily reduce ferricyanide to ferrocyanide.^30−32^ However, this reduced form cannot spontaneously react with ferric ammonium citrate at the provided dilution. The bond between iron and citrate cannot be spontaneously broken by the presence of ferrocyanide or bacteria but requires light activation. Our previous work has shown that using simply visible light illumination, provided by an artificial light source, the iron molecules uncouple from the citrate, freeing it for Prussian blue formation.^31,33,34^ If incubated with selected concentrations of antibiotics, bacterial susceptibility and/or resistance could also be determined with the same protocol. The sepsis kit is tested in the bacterial suspension of *E. coli* ATCC 25922 and *Staphylococcus capitis* (*Staph*) in a culture medium and blood. The interference of the components of the lysis buffer, the culture medium, and the blood in bacterial detection is studied. The kit is finally validated for susceptibility testing in spiked blood samples.

## Results and Discussion

### Working Principle of the Sepsis Kit

**Figure 1:**
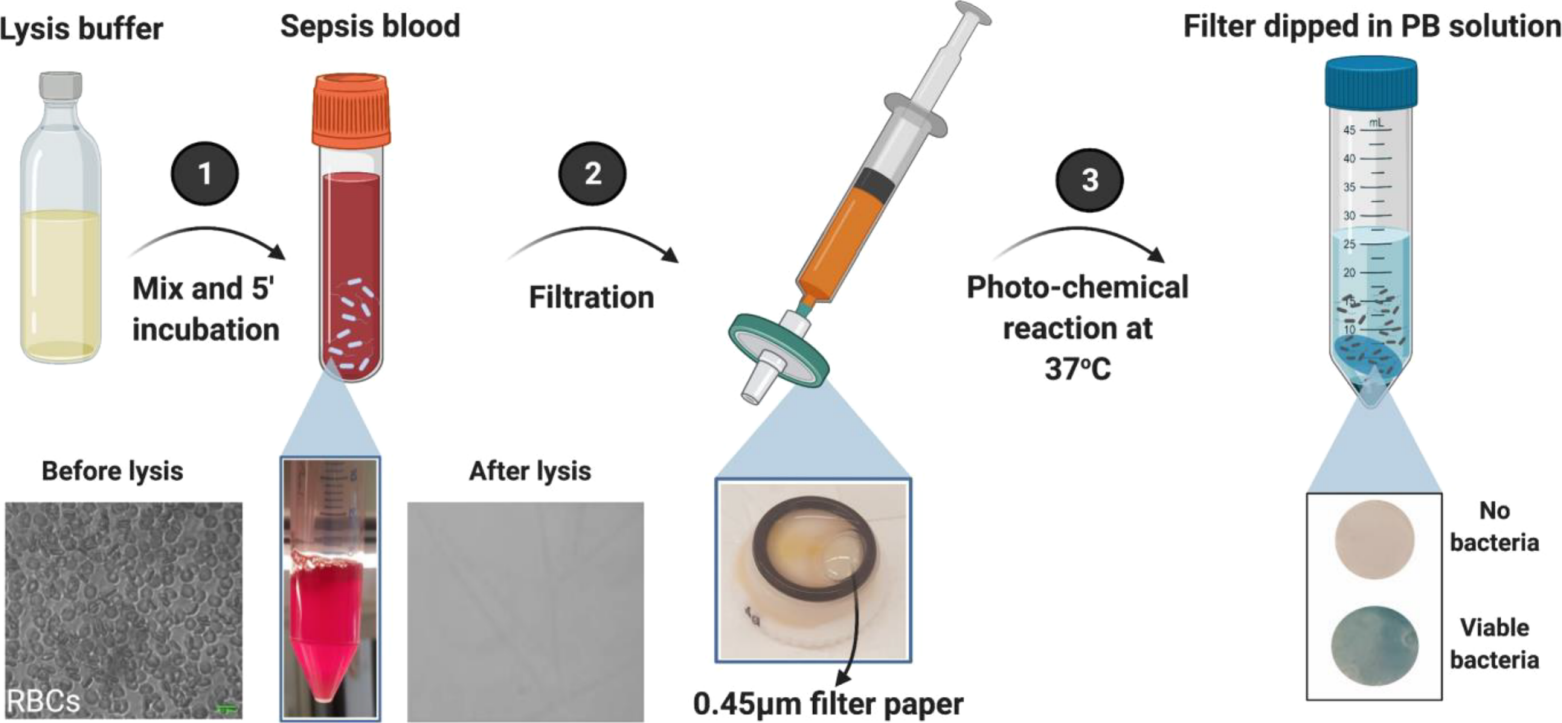
Schematic showing the working principle of selective isolation and detection of bacteria from whole blood. The method involves three steps. In the first step, lysis buffer is mixed with the whole blood sample (v/v) and incubated for 5 min with continuous mixing. The lysis buffer selectively ruptures blood cells keeping the bacteria completely viable. In step 2, the ruptured blood cells are filtered out through a simple filtration system. In step 3, bacteria captured on the filter paper are dipped into a culture medium with PB precursors. Filter paper containing viable bacteria turns blue, visible to the naked eye. Experimental results comparing the effect of lysis buffer on RBCs before and after exposure to lysis buffer and decreased viscosity of blood due to lysis buffer rupture are shown below step 1. Scale bar: 10 μm.

The method for isolating and detecting bacteria in whole blood involves two main stages: first, the selective lysis of blood cells by keeping the bacteria intact and viable, and second, the detection of viable bacteria using a photochemical amplification reaction producing intense blue color PB molecules. The working principle is illustrated in Figure 1 and summarized in three main steps. In the first one, whole blood samples containing bacteria were mixed with lysis buffer containing saponin and sodium cholate and incubated for 5 min with continuous stirring. The optimal concentration of lysis buffer was determined by evaluating the effect of different volume ratios of the lysis buffer mixture on whole blood samples (v/v). A ratio of blood to lysis buffer of 1:10 (v/v) was found optimal, aiming to lyse red blood cells (RBCs) with minimal dilution (see the Supporting Information, Figure S1). Regarding the incubation time, the effect of lysis buffer and its components over time on RBCs was evaluated by optical microscopy (Figure S2). RBCs treated with lysis buffer were completely ruptured after 5 min, as shown in the representative images included in Figure 1 (images below step 1). Lysed samples presented a less intense red color and lower viscosity due to the rupture of most RBCs initially present, being optimal for further microbial analysis.

In the second step, previously lysed blood samples were filtered out using a 0.45 μm cellulose filter, and the filter holder setup is shown in Figure 1. Intact bacteria were captured on the filtering membrane in a fast and simple process not requiring more than 1 min. After that, in step 3, the filter with viable bacteria was dipped into culture media containing the precursors of the photochemical reaction (PB solution), i.e., ferric citrate and ferricyanide, and incubated at 37 °C with exposure to continuous visible light irradiation. Bacterial proliferation over time turned both the filter paper and the solution into an intense blue color visible to the naked eye, which results from the metabolic production of PB molecules. This simple method provided a quick and selective way for bacterial isolation and detection directly from the whole blood sample, observable as a change of color to the naked eye in a few hours (<5 h for an initial change).

### Effect of Lysis Buffer on Lymphocytes and Platelets

**Figure 2:**
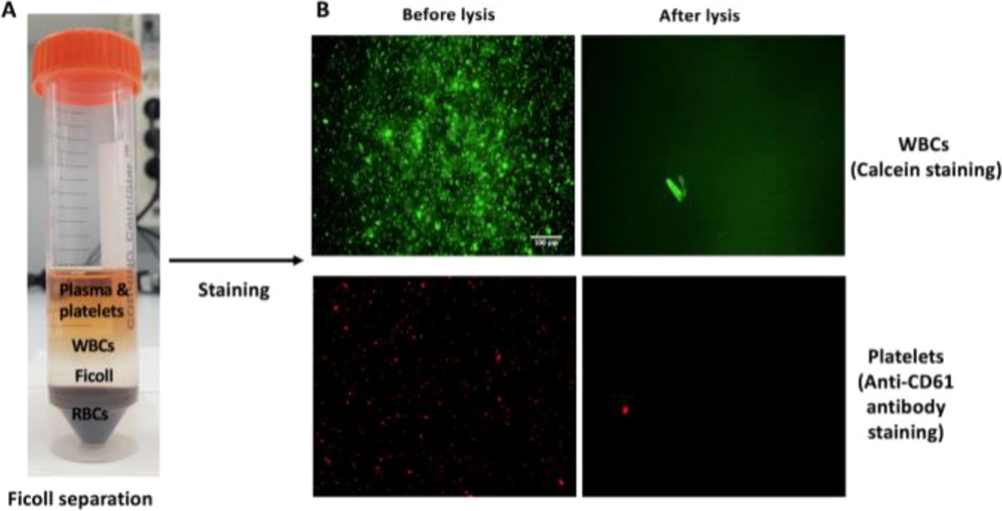
Effect of lysis buffer on WBCs and platelets. Ficoll density gradient separation was performed on whole blood to separate blood from its components. Isolated WBCs and platelets were stained using calcein dye and an anti-CD-61 antibody, respectively, comparing them before and after treatment with lysis buffer. WBCs and platelets were completely ruptured when treated with lysis buffer after 5 min. Scale bar:100 μm.

To evaluate the effect of the lysis buffer on other blood cell fractions, lymphocytes and platelets were isolated using the Ficoll density gradient-based separation method and analyzed independently (Figure 2A). Due to the difference in density, a Ficoll layer was formed between RBCs (collected at the bottom) and white blood cells (WBCs), while platelets and plasma were collected at the top layer. Isolated pure WBCs and platelets were exposed to the lysis buffer for 5 min. For visualization, WBCs and platelets were stained separately using calcein green AM dye and fluorescent-labeled anti-CD61 antibodies, respectively. The effect of lysis buffer was evident by fluorescence microscopy, where intact cells resulted in an intense green (for WBCs) or red (for platelets) color. As shown in Figure 2B, cell fluorescence disappeared, resulting from complete lysis of WBCs and platelets after 5 min of incubation.

### Effect of Lysis Buffer on Bacterial Viability

**Figure 3:**
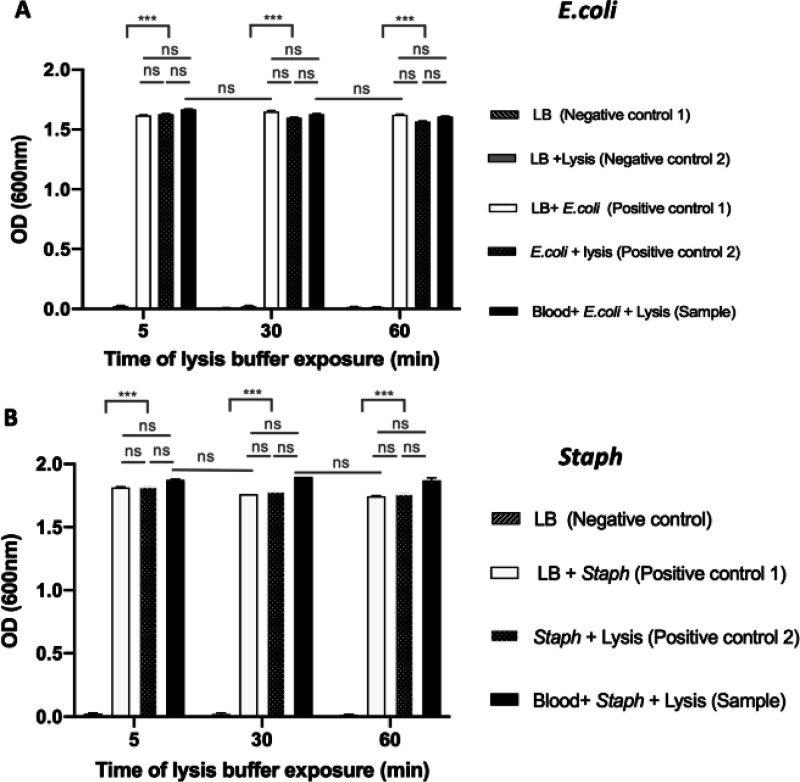
Effect of lysis buffer on the viability of bacteria. OD values were measured for different cases with two negative and positive controls (*n* = 3). These controls were compared with the sample (bacteria in blood and Lysis) for different lysis buffer exposure times (5, 30, and 60 min). (A,B) Graphs showing the OD measurements for *E. coli* and *Staph* in separate experiments, respectively. Similar OD values for the sample and positive controls for both *E. coli* and *Staph* show that 100% bacterial viability can be retained up to 1 h of lysis buffer exposure.

After verifying that among the lysis buffer components, only sodium cholate may compromise bacterial proliferation (Figure S3), the effect of lysis buffer on Gram-negative bacteria *E. coli* ATCC 25922 and Gram-positive bacteria *S. capitis* (*Staph*) was evaluated. High bacterial concentrations (10^6^ CFU/mL) were spiked into whole blood and exposed to lysis buffer for 5, 30, and 60 min (Figure 3). After exposure, bacteria were grown under optimal conditions overnight (17 h) in a culture medium and the OD at 600 nm was then determined as a semiquantitative measurement of bacterial proliferation. The following samples were analyzed for completeness of the experiment: (i) an LB medium alone (“LB”) and the LB medium mixed with lysis buffer (“LB + Lysis”) were used as negative controls (negative controls 1 and 2, respectively); (ii) bacteria spiked in the LB medium (“LB + *E. coli*” or “LB + *Staph*”) and bacteria exposed to lysis buffer (“*E. coli* + Lysis” or “*Staph* + Lysis”) were used as positive controls (positive controls 1 and 2, respectively); and bacteria spiked in whole blood and exposed to lysis buffer (“Blood + *E. coli* + Lysis” or “Blood + *Staph* + Lysis”) were used as the sample. Results presented in graphs (Figure 3A,B) show no significant differences (*p* > 0.05) between positive controls 1 and 2 and samples (*n* = 3) for all lysis buffer exposure times (5, 30, and 60 min). Negative controls were significantly different than positive ones and samples (*p* > 0.001) since no *E. coli* or *Staph* proliferation was recorded in negative controls, confirming no contamination of these controls. It may be concluded that the lysis buffer produced the selective disruption of the eukaryotic cell membrane, eliminating blood cell interferences in future blood analysis, but without compromising bacterial integrity since 100% viability was obtained considering the bacterial sample in the LB medium as proliferation reference. It is important to note here that even when sodium cholate alone presented important bactericidal activity, the correct combination of reagents in the final lysis buffer composition did not compromise bacterial viability, even when incubating the samples for long times of up to 1 h. This opens the possibility to expand the incubation time when necessary.

### Optimization of Viable Bacteria Detection through the Photochemical Formation of PB

**Figure 4:**
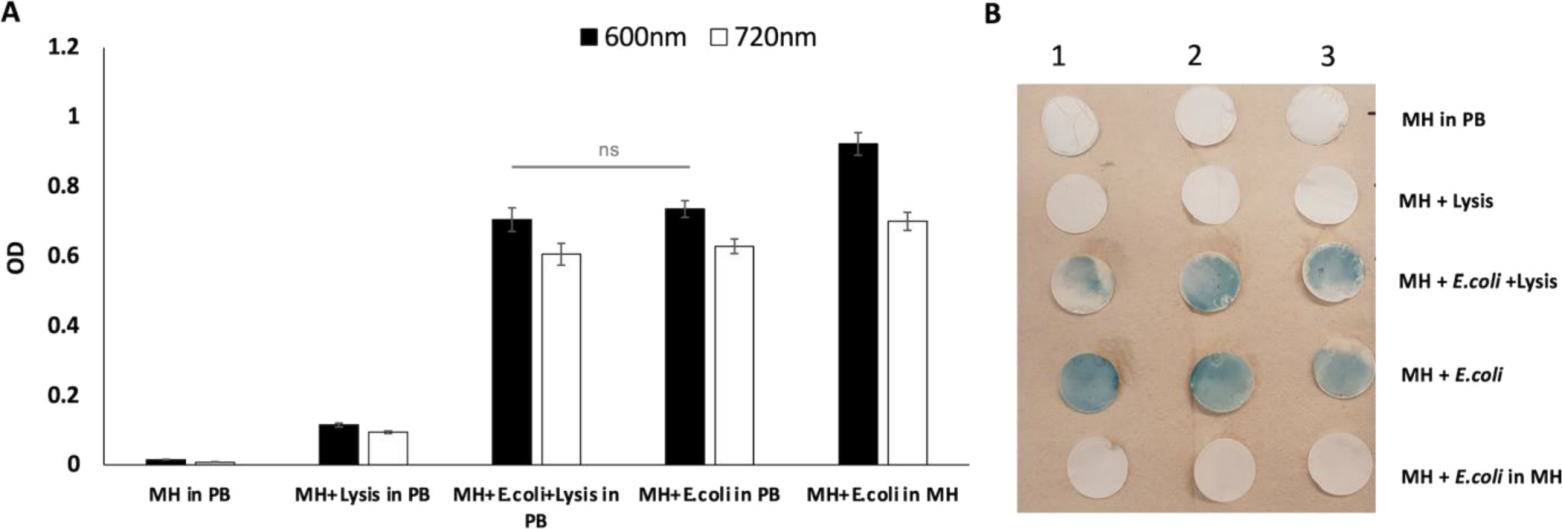
Detection of *E. coli* captured on filter paper after the selective cell lysis step using PB color formation. (A) Graph showing the OD values of PB solution-containing filter papers processed with negative controls (“MH in PB” and “MH + Lysis in PB”) and samples (“MH + *E. coli* in PB” and “MH + *E. coli* + lysis buffer in PB”) at 600 and 720 nm. “MH+ *E. coli* in MH” was used as a positive control. Similar OD values between the samples again showed no influence of lysis buffer on *E. coli* viability. (B) Prussian blue color was observed on the filter paper that was processed with samples containing *E. coli*, showing the need for bacterial viability for PB color formation (*n* = 3).

Detection of viable bacteria captured on the filter paper (step 2 in Figure 1) was achieved using the photochemical reaction, which resulted in the formation of PB molecules. Briefly, bacteria were incubated in a solution containing the PB precursors ammonium ferric citrate and potassium hexacyanoferrate. Viable bacteria metabolically reduce iron(III) complexes, initiating a photocatalytic cascade toward PB formation under constant illumination. Constant illumination was necessary for PB formation since it induced the photoactivation of ferric citrate and the release of free iron ions. The reaction of metabolically reduced iron(III) complexes with the photocatalytically released free iron ions resulted in the formation of PB molecules after short exposure times (<5 h). Before performing the experiments with blood, initial studies in bacterial culture media were conducted to optimize the bacterial detection protocol and to establish the assay conditions, e.g., sensitivity and limit of detection. *E. coli* samples containing 1000 CFU/mL were prepared in bacterial culture media and analyzed following the protocol described in Figure 1, by detecting the presence of bacteria on the filter paper and in the medium as an intense color change. For quantification, the OD of the medium was measured at 600 and 720 nm (Figure 4A), the latter corresponding to the absorption wavelength of PB molecules. In addition, images of the filter papers dipped in PB solution were captured after 17 h of incubation (Figure 4B). Filter papers processed with samples “MH + PB” and “MH + lysis buffer” dipped in PB solution were used as negative controls. Filter processed with samples having *E. coli* in MH dipped in MH media (“MH + *E. coli* in MH”) was used as a positive control of bacterial proliferation. As *E. coli* proliferated, blue color formation was observed in both the filter paper (containing viable bacteria) and the solution (as bacteria also grew in the solution) of the samples containing bacteria in PB precursor solution. Due to its high intensity, the color formation in the filter was even evident with the naked eye. OD values (*n* = 3) for filters processed with “MH + *E. coli*” and “MH + *E. coli* + lysis buffer” were not significantly different, confirming that the lysis buffer did not influence *E. coli* viability and proliferation (Figure 3). It is important to note that the negative controls showed low background OD and no blue color formation on the filter paper (Figure 3B), highlighting the need for viable bacteria for PB color formation.

As a final remark, the MH media used in this study may be substituted by conventional LB media since nonspecific interactions were observed between components of the PB precursor solution, LB media, and lysis buffer, as demonstrated in Figure S4 in the Supporting Information. The protocol was not only valid for Gram-negative bacteria but also produced positive responses in the case of Gram-positive bacteria (e.g., *Staphylococcus*; see Figure 6) and even mixtures of Gram-positive and Gram-negative bacteria (see the Supporting Information, Figure S5). This result confirmed the wide applicability of the assay that is sensitive to different bacterial strains and even complex bacterial mixtures. This opens the possibility to detect sepsis early without long genotypic studies.

### Evaluation of the Sensitivity and Response Time of the Sepsis Kit

**Figure 5:**
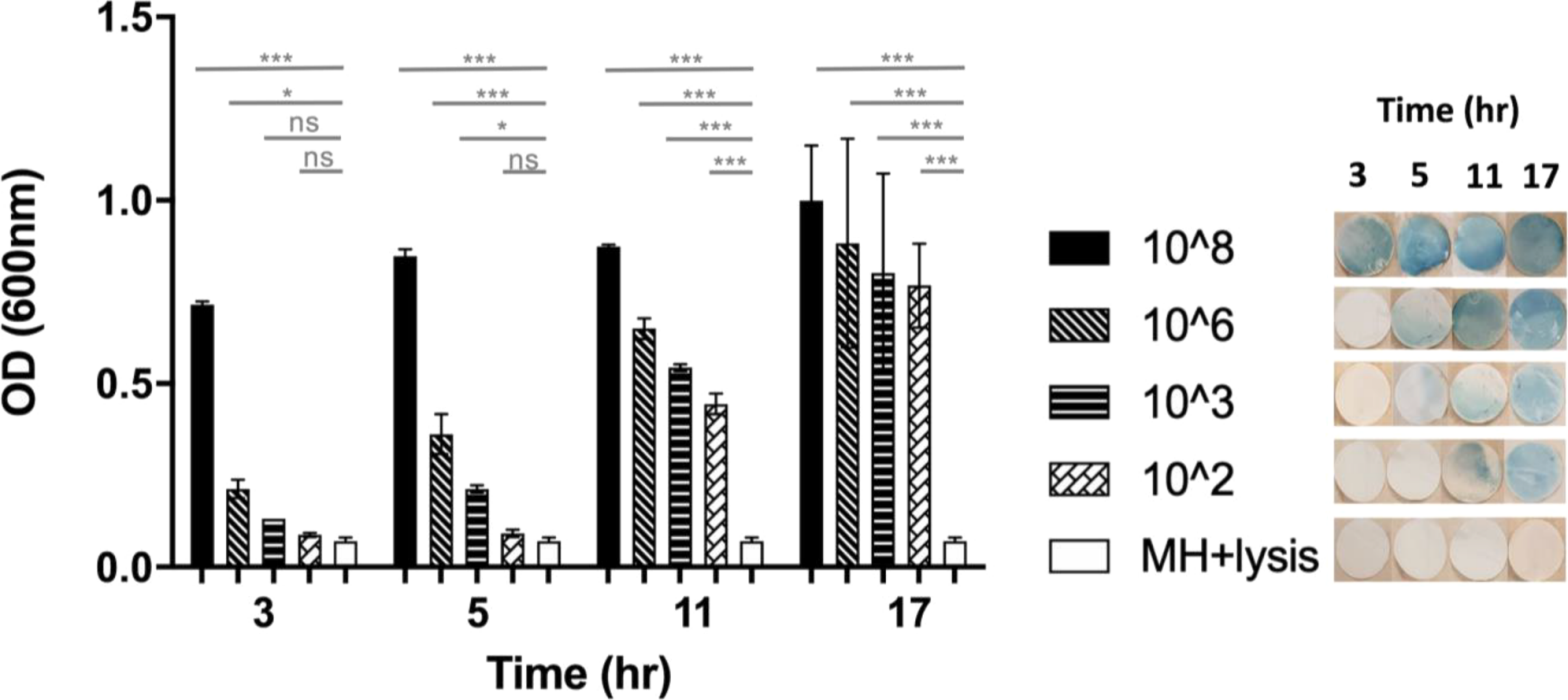
Dependence of time for PB color formation on bacterial concentration. The graph shows the OD values of the PB solution measured for four different concentrations of *E. coli* (10^8^, 10^6^, 10^3^, and 10^2^ CFU/mL), spiked into MH media in separate experiments (*n* = 3). Filtration was performed and PB color formation on the filter paper over four different time points (*t* = 3, 5, 11, and 17 h) was examined. It was observed that filter papers processed with *E. coli* concentrations of 10^8^, 10^6^, and 10^3^ CFU/mL showed blue color formation between *t* = 3 and 5 h. For a lower *E. coli* concentration (10^2^ CFU/mL), the time of PB color formation on the filter paper increased from 5 to 11 h.

As with all metabolic processes, the photochemically catalyzed production of PB by bacterial activity was a kinetic process depending on the concentration and metabolic activity of the culture. The time needed for the formation of PB color based on bacterial concentration was studied by spiking different concentrations of *E. coli* (10^8^ to 10^2^ CFU/mL) in MH media and analyzing them with the protocol detailed in Figure 1. “MH + lysis buffer” was used as a negative control. For each *E. coli* concentration, bacterial growth was determined by measuring the OD values at 600 nm (*n* = 3) of the PB solution at four time points (3, 5, 11, and 17 h), as shown in Figure 5. The filter papers were imaged for all the cases at the corresponding time points. For each of the cases, three experimental sets were performed (*n* = 3) and a representative paper sample is imaged in Figure 5. Taking the negative control sample as a reference, significant differences in the OD values were already obtained after 3 h of incubation by samples containing 10^6^ (*p* < 0.05) and 10^8^ CFU/mL (*p* < 0.001). Samples containing 10^3^ CFU/mL (*p* < 0.05) were significantly different after 5 h of incubation, while samples containing 10^2^ CFU/mL required 11 h to be significantly different than the negative controls (*p* < 0.001).Thus, the time necessary to detect bacteria in the sample with this method depended on the initial bacterial concentration, and concentrations above 10^3^ CFU/mL could be detected within the first 3 h of incubation, while samples containing 10^2^ CFU/mL require more than 5 h.

### Sepsis Detection in Bacterial-Spiked Whole Blood Samples

**Figure 6:**
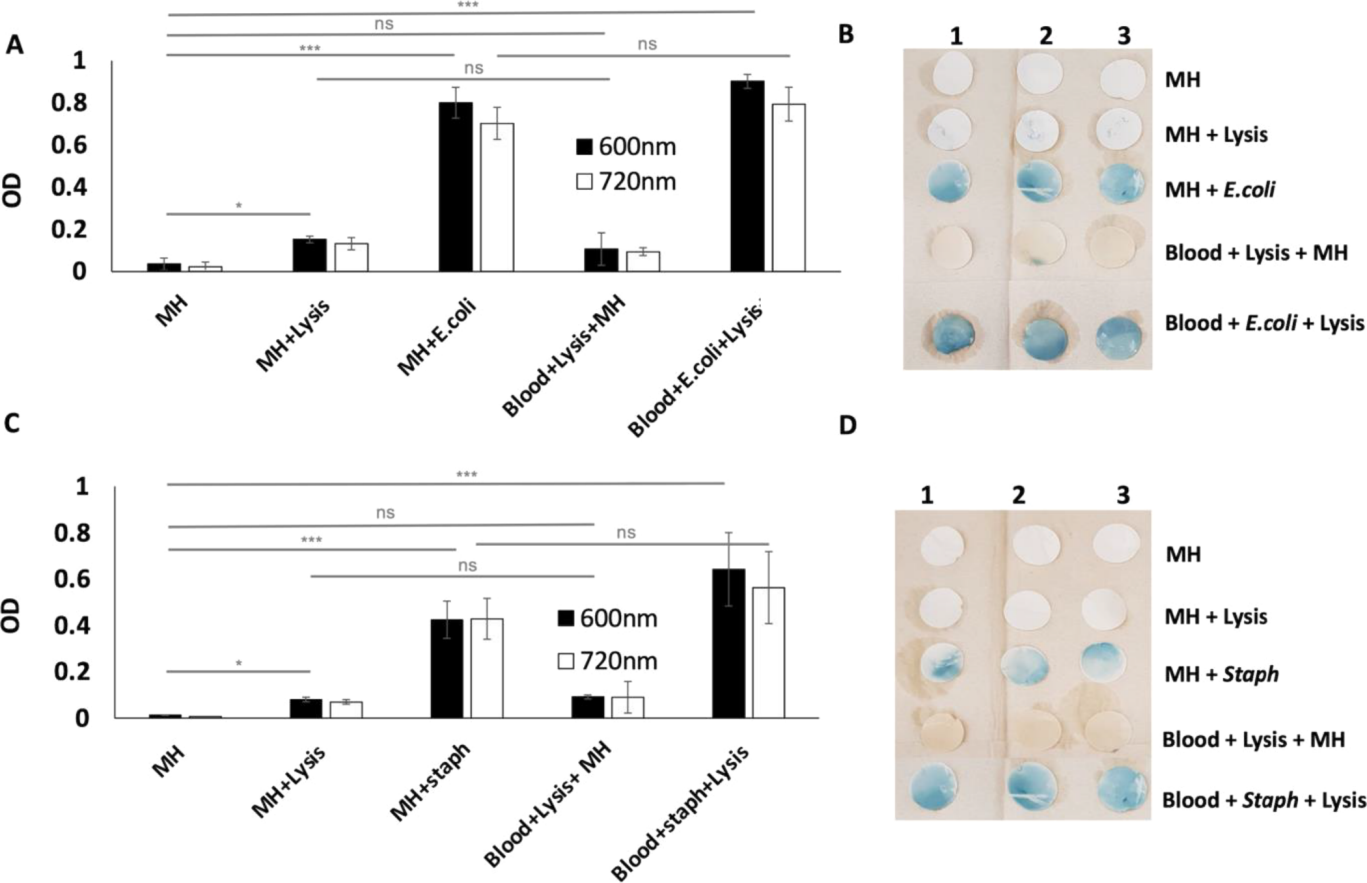
Detection of bacteria in spiked whole blood samples using PB color formation. A known concentration of bacteria (10^3^ CFU/mL) was spiked into whole blood and the protocol described in Figure 1 was performed. (A) Bar graph showing the OD values of the PB solution (into which the filter papers were dipped) measured after 17 h of incubation with *E. coli* (*n* = 3). Similar OD values of the PB solution on the filter paper processed with the positive control (“MH + *E. coli*”) and sample (“Blood + *E. coli* + Lysis”) showed no influence of blood and lysis buffer on bacteria viability. (B) Images of the filter papers taken after 17 h of incubation showed blue color formation on the filter papers processed with the positive control and sample having viable *E. coli* (*n* = 3). (C, D) OD values and the corresponding filter paper images for *Staph* are shown. Only those samples containing *Staph* dipped in PB solution showed PB formation.

A known concentration (i.e., 10^3^ CFU/mL) of *E. coli* and *Staph* was spiked into whole blood in separate experiments, and the capacity of the assay to detect bacteria in blood was evaluated by following the protocol described in Figure 1. The graph in Figure 6A shows the OD quantification of the PB solution at 600 and 720 nm, measured for the positive control (“MH + *E. coli*”), negative controls (“MH”, “MH + Lysis”, and “Blood + Lysis + MH”), and the sample (“Blood + *E. coli* + Lysis”) with *n* = 3. Additionally, the corresponding images of the filter papers dipped in the PB solution were also acquired after 17 h (Figure 6B). No significant differences were obtained when comparing the OD measurements at 600 nm for positive controls and samples (*p* > 0.05), which confirmed the low influence of blood and lysis buffer on bacterial viability. Higher OD values at 720 nm and PB color formation were observed only on the filter papers corresponding to positive controls and samples, again confirming the need for viable *E. coli* to produce a detectable PB color formation. No statistically significant differences (*p* > 0.05) were observed between most of the negative controls, and the small difference between the “MH” and “MH + Lysis” samples, which was statistically significant (*p* < 0.05), may be attributed to the absorption/scattering of components of the lysis buffer. Similar results were obtained in the case of blood samples spiked with Gram-positive bacteria, i.e., *Staph*, as shown in Figure 6C,D (OD values and filter paper images, respectively). These results confirmed the low or null cross-reactivity between the components of the precursor solution and those present in the blood samples.

### Antibiotic Susceptibility Testing

**Figure 7:**
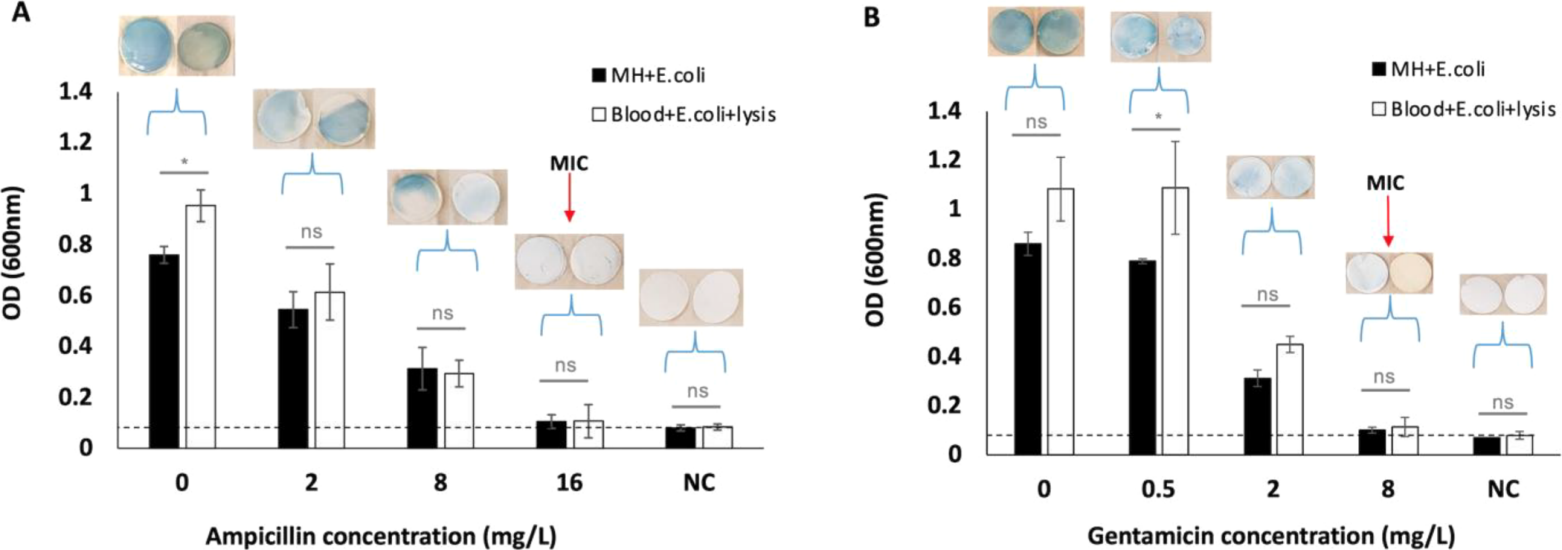
Antibiotic susceptibility testing using PB color formation. The lysis buffer-based sample preparation method combined with detection using PB formation was used to perform antibiotic susceptibility testing using different concentrations of ampicillin and gentamicin for *E. coli* ATCC 25922. Images of the PB color formation on the filter paper and the corresponding OD values measured after 17 h of incubation are shown in the graph. For both antibiotics, it was observed that the OD values and the intensity of blue color formation on the filter paper decreased with an increase in antibiotic concentration (*n* = 3). (A, B) MIC of ampicillin and gentamicin was determined to be 16 and 8 mg/L, respectively, which agreed with the MIC range provided by the EUCAST.

Due to the severity of blood infections, wide-spectrum antibiotics are prescribed as the first-choice treatment for sepsis before any genotypic or phenotypic analysis is performed. This allows fast decision-making with a high probability of failure by, e.g., the presence of resistant bacteria or due to inappropriate selection of antibiotics. One way to solve this issue is by performing fast antibiotic susceptibility testing directly in blood samples, without culture or purification of the bacteria. This is possible with the protocol detailed in Figure 1, by incubating viable blood bacteria captured in the filter paper with the antibiotic and performing a susceptibility test in less than 5 h. The minimum inhibitory concentration (MIC) was determined by following the protocol provided by the European Committee on Antimicrobial Susceptibility Testing (EUCAST).^35^ MIC was selected for the concentration of the antibiotics at which low or null PB color formation was observed. Two antibiotics were tested in this case, ampicillin and gentamicin, and the experimental values were compared with those tabulated by the EUCAST for *E. coli* ATCC 25922, i.e., 2–8 mg/L for ampicillin and 0.25–1 mg/L for gentamicin. Experimentally, a known concentration of *E. coli* (10^8^ CFU/mL, recommended McFarland 0.5 standard concentration) was spiked into whole blood and the protocol described in Figure 1 was followed. Different concentrations of ampicillin (0–16 mg/L) and gentamicin (0–8 mg/L) were added to the PB precursor solution during incubation (step 3 of Figure 1). “MH + Lysis”, “MH + *E. coli*”, and “Blood + *E. coli* + Lysis” were used as the negative control (NC), positive control (0 mg/L), and sample, respectively. In addition to taking the images of the filter paper, OD values of the PB solution were also acquired at 600 nm after 17 h of incubation by comparison with standard MIC protocols. The bar graph in Figure 7 shows the average value and standard deviation of the measured OD values of three experimental sets (*n* = 3) for each antibiotic concentration under study. Filter images in the figure correspond to representative samples. As expected, OD values for the positive control and sample decreased when increasing antibiotic concentration (Figure 7A,B). No significant differences were obtained when comparing absorbance values at 600 and 720 nm, as illustrated in the Supporting Information (Figure S6 for ampicillin and Figure S8 for gentamicin), confirming that both wavelengths are equally valid to report on bacterial activity through this assay. In agreement with previous results, the intensity of blue color formation on the filter paper also decreased with the increasing antibiotic concentration, signifying the loss of bacterial viability and metabolic activity at high antibiotic concentrations. Considering both OD and blue color formation, MIC values for ampicillin and gentamicin of 16 and 8 mg/L, respectively, were obtained. These values were slightly higher than those provided by the EUCAST, which are in ranges of 2–8 and 0.25–1 mg/L for ampicillin and gentamicin, respectively. As already reported by Mouton *et al*.,^36^ a small difference in the MIC magnitude can be obtained when comparing new approaches with those established by the EUCAST, and even the EUCAST states that an MIC value differing 2-fold from the standard method should be considered reliable. In this case, the cyanotype-based reaction was two orders of magnitude more sensitive than broth microdilution, being able to detect 10^3^ CFU/mL within 3–4 h of the reaction. Due to this enhanced sensitivity, the MIC values provided by this assay were slightly higher than those tabulated, although very close to the 2-fold limit stablished by the EUCAST. The current method may be additionally improved by quantifying blue color formation with spectroscopic systems instead of by visual inspection, as performed in the current work.

As a proof of principle, the experiment was repeated with a low concentration of *E. coli* (10^3^ CFU/mL) and the MIC for ampicillin was determined to be 16 mg/L as shown in Figure S7. Although the measurements were performed after 17 h, the initial blue color formation on the filter paper was already observed after 3 h of incubation, thus having the possibility to provide MIC in a much shorter time.

This result demonstrates the applicability of this selective cell lysis and photochemical reaction for fast antibiotic susceptibility testing directly in blood samples, being able to determine the most suitable treatment in sepsis in a few hours, even when not knowing the specific bacterial strain responsible for the infection.

## Conclusions

In this paper, we present a novel sepsis kit, where a selective cell lysis-based sample preparation method is combined with a highly sensitive photochemical reaction capable of reporting the presence of bacterial infection in blood samples within 5 h for 10^3^ CFU/mL bacterial concentration through a simple color change. We demonstrate this by initially characterizing the selective cell lysis buffer to specifically rupture blood cells in 5 to 10 min, without compromising bacterial integrity and activity (i.e,. 100% viable). Using a simple syringe-based filtration setup, viable bacteria are retained in a filter paper, while lysed blood cells are completely washed out, not interfering with the subsequent analysis. Filter papers containing blood bacteria are then incubated in the PB precursor solution in an MH medium under continuous irradiation. This incubation starts a photocatalytic and metabolically mediated chemical reaction, resulting in the formation of intense blue PB molecules only when living and metabolically active bacteria are retained in the filter (negative control samples without bacteria remain white). The detection process is sensitive to both Gram-negative and Gram-positive bacteria and even to a mixture of different bacterial strains.

Furthermore, as a proof of concept, we show that this method can also be used to perform antibiotic susceptibility testing by exposing the filter paper having bacteria to different concentrations of antibiotics, which was tested using ampicillin and gentamicin, and providing similar results as gold standards after 3 h of incubation. The current plating method needs 2 to 3 days from sample preparation to detect bacteria in sepsis blood samples. As a short time to diagnose sepsis is very crucial, we believe that this new method has great potential in the clinical settings to quickly select positive patient samples from the negative ones, which helps in prescribing appropriate antibiotics, thus reducing antibiotic abuse. Furthermore, this simple syringe-based sample preparation to color change-based bacterial detection has very high potential in resource-limited settings toward sepsis diagnostics, as it is easy to use and does not demand the need for expensive equipment.

## Methods

### Lysis Buffer

Lysis buffer was prepared by mixing sodium cholate hydrate (MW: 430.55 g, Sigma-Aldrich) and saponin (1.015–1.020 g/mL at 20 °C, Sigma-Aldrich). Concentrations of 4% sodium cholate and 2% saponin were dissolved in 1X phosphate-buffered saline (PBS tablets, pH: −7.2 to 7.6, Sigma-Aldrich) separately. Equal volumes of each were mixed to obtain a final pH of 6.5, which was used as a lysis buffer for the sample preparation method. The final concentration in the lysis buffer is 1% saponin and 2% sodium cholate.

### Prussian Blue

Colorimetric detection was done using Prussian blue color formation. Ammonium ferric citrate (2.5 mM; iron content of 16.5 to 18.5%, Sigma-Aldrich) and potassium hexacyanoferrate (0.625 mM; MW = 329.24 g/mol) were prepared using Mueller–Hinton (MH) media. The final solution was adjusted to a pH of 6.5.

### Antibiotics

Ampicillin powder (MW = 349.4 g/mol) and gentamicin sulfate salt white powder (potency: 600 μg of gentamicin per mg) were ordered from Sigma-Aldrich.

### Culture Media

Luria–Bertani (LB) broth miller (pH range: 6.8 to 7.2) and MH broth 2 (final pH: 7.3. ± 0.2 at 25 °C) were ordered from Sigma-Aldrich. For all the experiments with MH media, the pH of the final MH liquid broth was adjusted to 6.5 using 0.1 M HCl.

### Characterization and Optimization of Lysis Buffer Activity

For characterizing the lysis buffer, a known concentration of *E. coli* ATCC 25922 strain was spiked into LB media and was mixed with lysis buffer (1:10 v/v). The mixture was filtered using a 10 mL B.D plastic Luer-lock syringe by placing a 0.45 μm nitrocellulose filter paper (MF: Millipore membrane filter mixed with cellulose esters, Sigma-Aldrich) with 25 mm diameter in a filter holder (easy-pressure syringe filter holder, 25 mm, Pall Laboratory). Translucent polyethylene terephthalate (PET) filters (SABEU plastics and membrane technology) with 0.4 μm pore size with 25 mm diameter were used for PET-based experiments. Filter papers with captured *E. coli* were dipped into a freshly autoclaved LB/MH broth and were incubated at 37 °C overnight (17 h). After overnight culture, optical density (OD) measurements were performed using a spectrophotometer (Spectromax M series, molecular devices) at OD = 600 nm.

### Ficoll Separation

Standard Ficoll density gradient separation was performed on 1 mL of whole blood to separate blood cells from its components. Whole blood was diluted in 1:5 v/v using 1X PBS and was added to 7.5 mL of Ficoll solution. The mixture was centrifuged for 30 min at 400*g* without break. Pure lymphocytes were carefully removed and centrifuged for 10 min at 350*g*. The supernatant containing PBS was discarded and the pellet was resuspended in PBS. Isolated lymphocytes were stained with calcein green AM dye for visualization and incubated with the lysis buffer at room temperature for 15 min in the dark to avoid photobleaching of the fluorescent dye.

### Eukaryotic Cell Staining

WBC staining was performed using CellTrace calcein green AM dye (Thermo Fischer Scientific). Ficoll-separated pure WBCs were stained by adding 1 μL of calcein green AM dye to 1 mL of the sample and incubated at room temperature for 15 min in the dark and viewed using a fluorescence microscope before exposing to the lysis buffer. To visualize platelets, Ficoll-separated plates were centrifuged again at 1200 rpm for 5 min and the pellet was resuspended in 100 μL of 1X PBS. Platelets were stained with a 10 μg/mL final concentration of Alexa Fluor 647 anti-human CD61 antibody. This was incubated in the dark for 25 min at 4 °C. This mixture was centrifuged at 1200 rpm for 5 min, and the supernatant was discarded. The pellet was resuspended in 100 μL of 1X PBS and then visualized using a fluorescence microscope before exposure to the lysis buffer.

### Colorimetric Bacterial Detection

An LED spotlight (15 W, 6400 K, 85–256 V Vanessa-15) was used as a light source (a range of visible light wavelength, mostly in blue color) to induce the photocatalytic reaction. In addition to qualitative detection based on color change, we also measured the absorbance by spectrometry to semiquantitatively measure bacterial concentration based on the PB color formation at 600 and 720 nm, respectively. Here, 720 nm is the wavelength specific to the absorbance of PB.^31,34^ Captured bacteria on the filter paper were detected by colorimetric analysis. After the filtration step, filter papers were dipped into 5 mL of a solution containing 625 μL each of 2.5 mM ammonium ferric citrate and 0.625 mM potassium hexacyanoferrate in MH media (referred to as PB solution). Two negative controls and two samples were used in the experiment to demonstrate the formation of PB color in the presence of bacteria. Negative controls included (1) MH media and (2) MH media mixed with lysis buffer (1:10 v/v). The two samples included were (3) *E. coli* in MH and (4) *E. coli* in MH exposed to the lysis buffer. Filter papers were dipped into PB solution at 37 °C for 17 h (overnight) and continuously exposed to light. After overnight culture, OD measurements were performed at 600 and 720 nm.

### Blood Samples Analysis

For the blood sample analysis, fresh blood samples were collected from healthy blood donors from the blood center GeBlod, Stockholm, Sweden, in EDTA tubes. Experiments were performed on the day of blood collection. No other blood pretreatment step was performed. One milliliter of the whole blood was mixed with lysis buffer and incubated for 5 min at room temperature with continuous stirring. Different ratios of whole blood to lysis buffer volume were tried, ranging from 1:1 to 1:10 (v/v).

### Antibiotic Susceptibility Testing

Different concentrations of antibiotics ampicillin and gentamicin were used in the experiments. A stock solution (5.12 mg/mL) was prepared by dissolving the antibiotic powder in Milli-Q water. The desired concentrations of antibiotics were selected using the EUCAST (European Committee on Antimicrobial susceptibility testing) method. The required concentration of antibiotics was further obtained by diluting the stock using MH media. After capturing the bacteria on the filter paper using the process explained in Figure 1, the filter paper was dipped into a solution containing 2.5 mL of antibiotics and 625 μL each of 2.5 mM ammonium ferric citrate and 0.625 mM potassium hexacyanoferrate and made up to a total volume of 5 mL using MH media. This setup was finally incubated in the presence of a light source in a 37 °C incubator overnight.

### Data and Statistical Analysis

Statistical analysis was performed using GraphPad Prism 6 software (RRID:SCR_002798; GraphPad Software, La Jolla, CA, USA). All results presented in the article are expressed as mean ± standard deviation. One- or two-way ANOVA followed by Bonferroni’s post-hoc correction was, respectively, used to compare one or multiple variables. Statistical analysis was performed only when each group size was at least *n* = 3 independent variables. The threshold for statistical significance was *p* < 0.05 throughout.

## Supporting Information Available

Effect of concentration of lysis buffer on whole blood (Figure S1); effect of lysis buffer and its components on blood cells over time (Figure S2 with GIF); effect of components of lysis buffer on *E. coli* viability in the blood (Figure S3); detection of *E. coli* in the LB medium by PB color formation (Figure S4); detection of the Gram-negative and Gram-positive mixture using PB formation (Figure S5); antibiotic susceptibility testing using ampicillin at 720 nm (Figure S6); antibiotic susceptibility testing using ampicillin for low concentration of *E.coli* (Figure S7); antibiotic susceptibility testing using gentamicin at 720 nm (Figure S8) (PDF)
