# Supplementary material for: Toward Rapid Detection of Viable Bacteria in Whole Blood for Early Sepsis Diagnostics and Susceptibility Testing
Source: ACS Sens. 2021 Aug 19;6(9):3357–66. doi: 10.1021/acssensors.1c01219 (PMC8477386; doi:10.1021/acssensors.1c01219)
Supplement: Supplementary file 1 — se1c01219_si_001.pdf [file se1c01219_si_001.pdf]

Supplementary information

Toward Rapid Detection of Viable Bacteria in Whole Blood for Early Sepsis Diagnostics and Susceptibility testing

Sharath Narayana Iyengar[‡12],Jiri Dietvorst [‡3], Amparo Ferrer-Vilanova[3], Gonzalo Guirado[4], Xavier Muñoz-Berbel[*3], Aman Russom[*12]

[1]Division of Nanobiotechnology, Department of Protein science, Science for life laboratory, KTH Royal Institute of technology, 17165 Stockholm, Sweden

[2]AIMES - Center for the Advancement of Integrated Medical and Engineering Sciences at Karolinska Institutet and KTH Royal Institute of Technology, Stockholm, 17165, Sweden

Email: aman@kth.se

[3]Instituto de Microelectrónica de Barcelona (IMB-CNM, CSIC),Campus Universitat Autónoma de Barcelona, Cerdanyola del Vallès, 08193 Barcelona, Spain

Email: xavier.munoz@imb-cnm.csic.es

[4]Department de Química, Universitat Autònoma de Barcelona, 08193, Cerdanyala del Vallès, Barcelona, Spain

Effect of concentration of lysis buffer

Incubation of blood samples with the lysis buffer implies the dilution of the already low bacterial concentration in blood, although a too low concentration of buffer may reduce the efficiency of cell lysis. Eight different dilutions from 1:2 to 1:10 (blood : buffer ratio in v/v) were evaluated after 5min of incubation and analyzed by microscopy. The ratio mixture is defined based on volume to volume ratio (v/v). For instance, 1:2 denotes 1mL of whole blood mixed with 2mL of lysis buffer. Volume of blood was always kept constant to 1mL while the volume of lysis buffer was varied. Only in the 1:2 sample, some intact bacteria were still observed in the microscopic preparation (Fig.S1), suggesting that the cut off concentration ensuring complete blood cell lysis was in between 1:2 and 1:4. The incubation of blood samples in a 1:4 dilution for 5min was selected as the optimal lysis protocol. However, 1:10 dilution was used in further experiments to retain the highest concentration of lysis buffer.

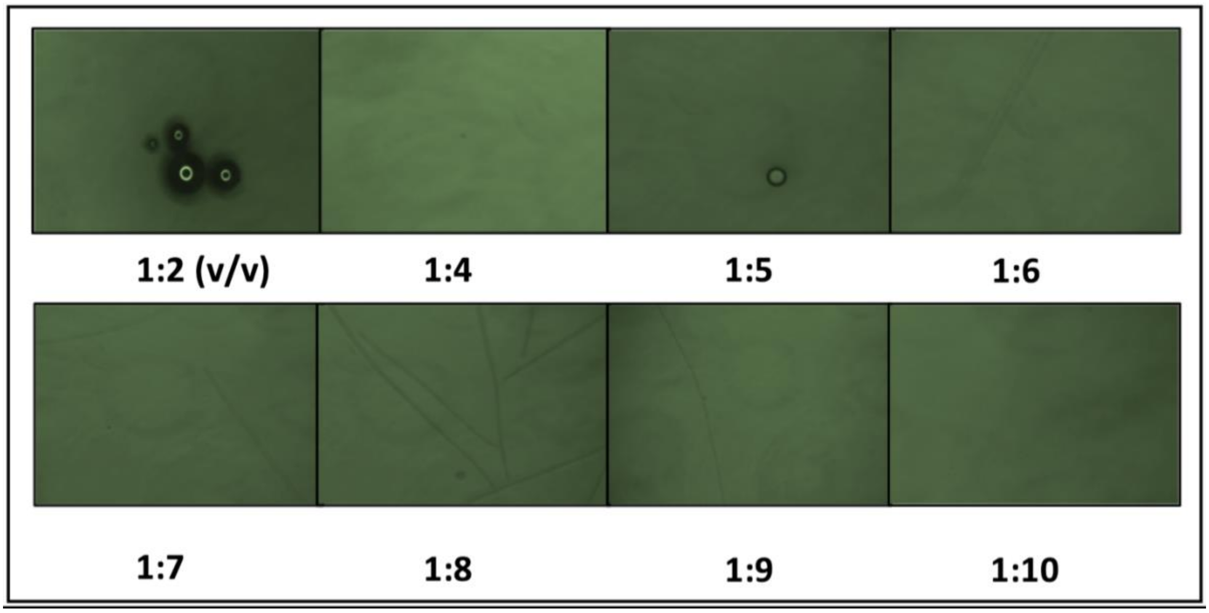

Fig.S1. *Effect of concentration of lysis buffer on whole blood. Different volume to volume ratio of whole blood to lysis buffer from 1:2 to 1:10 was tested. The results showed that there were few blood cells still intact in 1:2 case while cases above 1:4 had completely ruptured all blood cells. The cut off ratio was thus found to be between 1:2 to 1:4.*

Effect of lysis buffer and its components on blood cells over time

The effect of lysis buffer and its components on the blood cells were investigated in separate experiments over time and bright field images were captured. After exposing to 2% saponin, few blood cells (RBCs) were still observed even after 1hr of exposure. In addition, few blood cell clumps were also observed in few cases after 1hr (highlighted in red box). On the other hand, all the blood we completely ruptured after exposing to 4% sodium cholate and lysis buffer after 110s and 100s respectively.

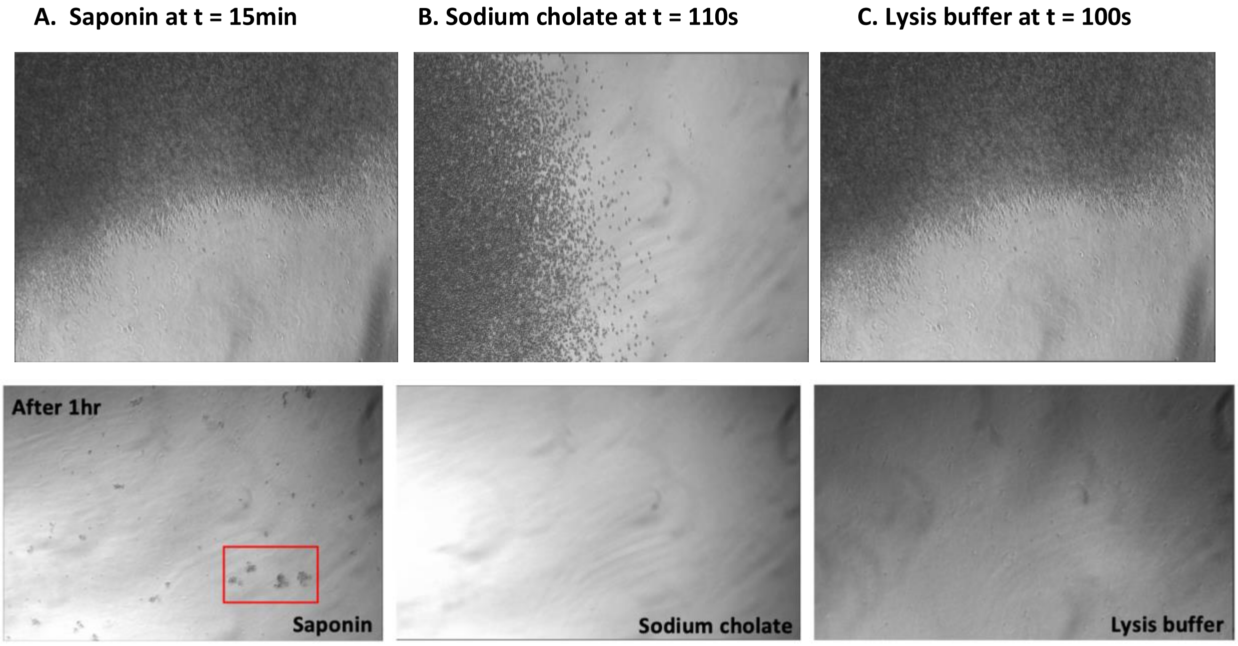

Fig.S2. *Lysis of blood cells over time. Whole blood was treated with 2% saponin, 4% sodium cholate and lysis buffer, in separate experiments in 1:10 (v/v) ratio on a well plate and bright field images (10X magnification) were taken from 0 to 60min. It was observed that blood cells were not completely ruptured until 15min of saponin exposure shown as in GIF of Fig.S1A. After 1hr of saponin exposure, we still observed few blood cells and some cells formed clumps (highlighted using a red box). On the other hand, complete rupture of blood cells were observed by exposing to sodium cholate and lysis buffer in 110s and 100s, respectively as shown in Fig.S1B and Fig.S1C. Scale bar: 500µm (B) Fast forward (100X) video showing rupture of blood cells when exposed to lysis buffer.*

Effect of components of lysis buffer on *E.coli* viability in blood

Effect of lysis buffer and components of lysis buffer were compared to study their effect on bacterial viability over time in blood. Initial concentration of 10^3 CFU/mL *E.coli* was spiked into whole blood and exposed to 2% sodium cholate, 1% saponin and lysis buffer separately and incubated over time (0 to 4hr) at room temperature. No lysis buffer condition was used as negative control (*E.coli* in PBS). After every hour, agar plating was performed and colonies were counted after 17hr of incubation at 37°C (n=3). Statistical analysis revealed no significant (ns) difference for all the cases up to 1hr of incubation. As the incubation time was increased no significant difference was obtained between *E.coli* in MH (No lysis) and *E.coli* exposed to saponin. This showed that saponin did not affect the *E.coli* viability over time. On the other hand, we observed a decrease in *E.coli* count which reduced to zero after exposing to sodium cholate and a significant difference; p<0.001 (***) was observed for sodium cholate after 2hr of incubation signifying that sodium cholate when exposed alone, kills bacteria. However, mixing saponin and sodium cholate in the lysis buffer revived the *E.coli* viability over time, signifying the importance of lysis buffer on bacterial viability with a value of p<0.01 (**).

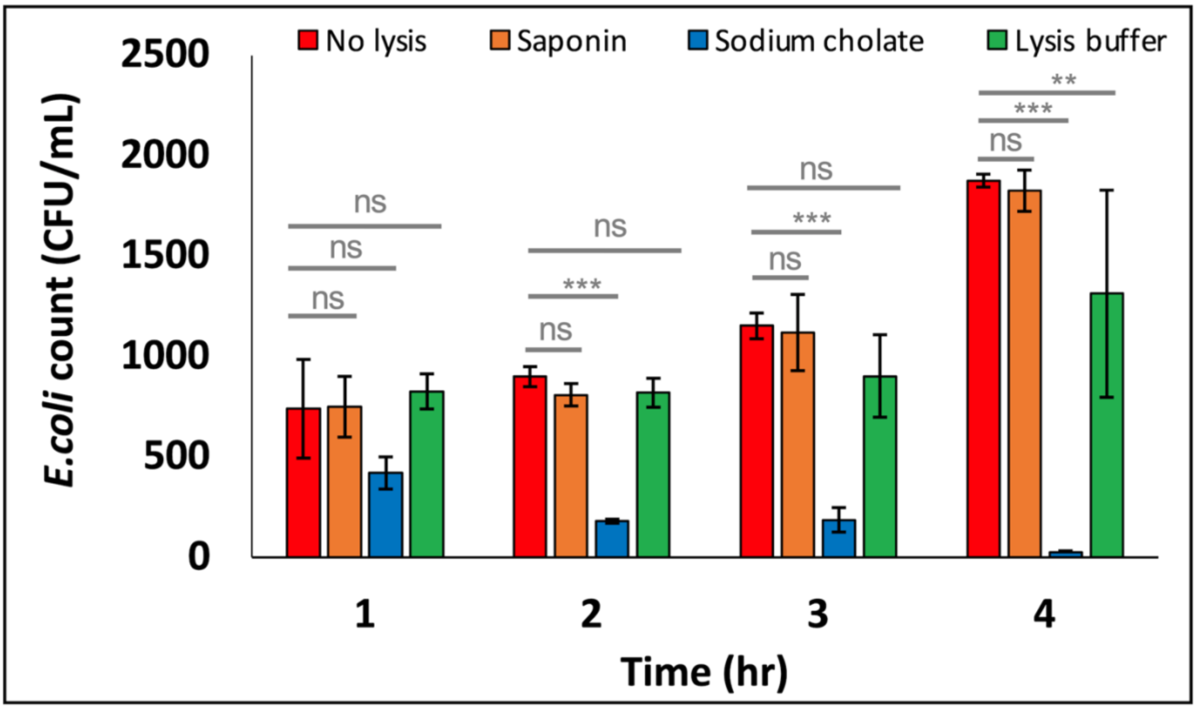

Fig.S3. *Effect of lysis buffer and its components on viability of E.coli in blood over time. 1% saponin, 2% sodium cholate and lysis buffer were exposed to E.coli spiked in blood. Agar plating was performed over time from 1hr to 4hr for all the cases (n=3). It was observed that after 2hr of exposure to sodium cholate, there was a decrease in E.coli growth and no growth was observed after 4hr of exposure. However, for both saponin and lysis buffer, the viability of E.coli was retained for all the time points.*

Detection of *E.coli* in LB using PB color formation

Initial experiments were performed by spiking *E.coli* in LB media for optimizing the PB based detection of bacteria. Bar plot in Fig.S3 shows the OD values obtained after incubating in PB solution for 17hr from four different samples (n=3) after performing the protocol described in Fig.4. Samples ‘LB + PB’ and ‘LB + lysis buffer’ were used as negative controls. The three samples containing bacteria had a bacterial concentration of 10^6 CFU/mL. The negative control samples having a combination of ‘LB + PB’ and the sample having ‘LB + lysis buffer’ showed a high background OD values of 1 and 3 A.U respectively with a significant difference; p<0.001 (***). This is due to some reaction between components present in LB, PB and lysis

buffer solution which is still under evaluation. The sample having Lysis buffer, PB and *E.coli* also showed a very similar O.D value of 3.5 A.U which is not a significant change (ns) in value to differentiate from 'LB + Lysis buffer + PB'. As a result, LB media was replaced by MH (Mueller-Hinton) media for future experiments.

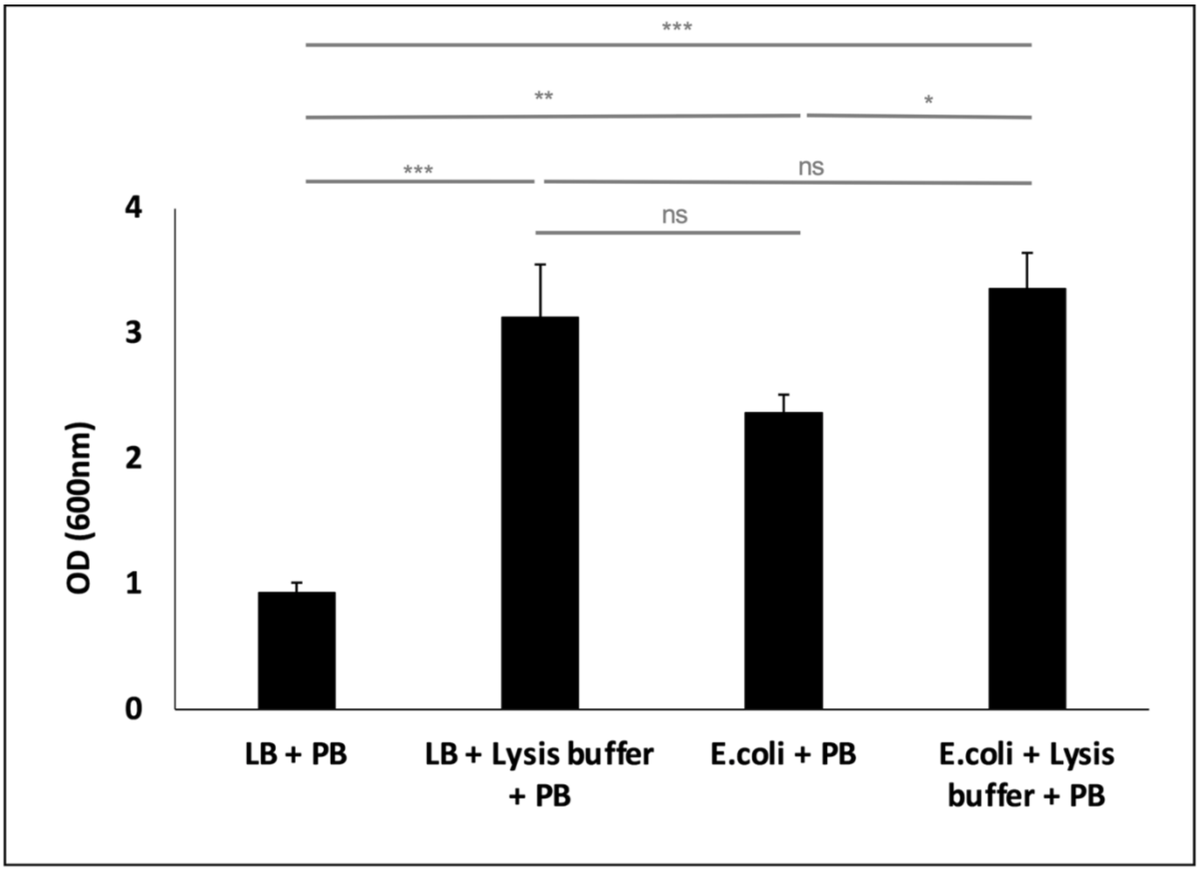

Fig.S4. *Detection of E.coli in LB using PB color formation. Graph showing the O.D values measured after 17hr from different samples at 600nm using LB media (n=3). O.D values in all the experiments are measured from the solution into which the filter paper is dipped and incubated in. A huge background O.D value of 1 and 3 A.U were observed for negative controls 'LB + PB' and 'LB + lysis buffer + PB' making it challenging to differentiate from a sample having bacteria.*

Detection of gram negative and gram positive mixture using PB formation

To understand the change in viability in a sample containing mixture of bacteria, two different bacterial species were spiked in MH media. Equal concentration (1000 CFU/mL) of gram negative (*E.coli*) and gram positive (*Staphylococcus Capitis*) and absorbance was measured after incubating the filter paper for 17hr in PB solution as shown in graph of Fig.7A (n=3). It was observed that the absorbance was similar for the samples with and without lysis buffer exposure (''MH + *E.coli* + *Staph*'' and ''MH + *E.coli* + *Staph*+ Lysis'') signifying very low effect of lysis buffer on bacterial viability. In addition, the filter paper were taken out from the tubes and were compared to see the PB color formed on the filter paper as shown in Fig.7B It was observed that the sample containing bacteria formed blue coloration on the filter paper confirming the need to bacterial metabolism for PB formation.

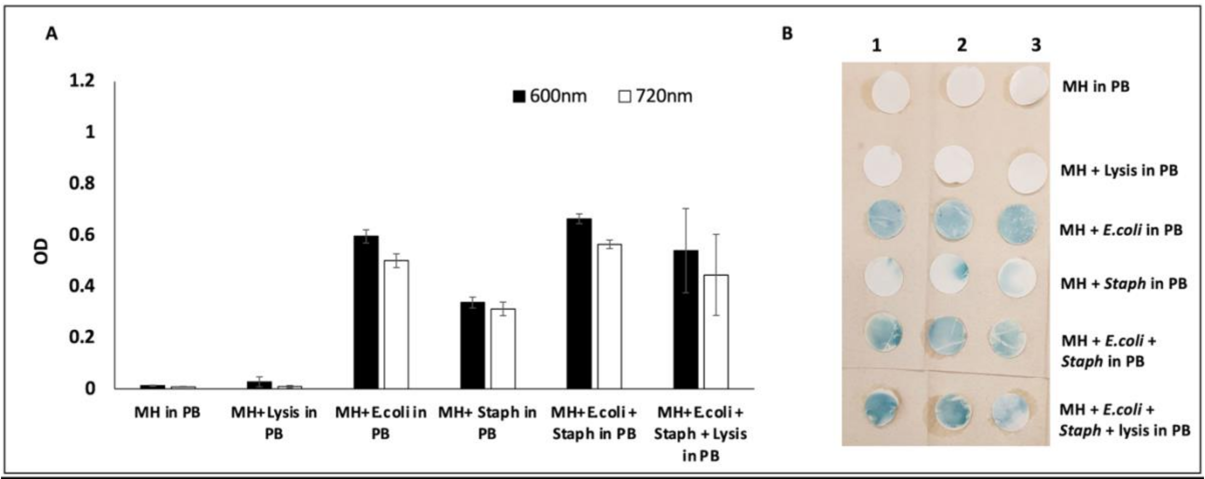

Fig.S5. *Detection of a mixture of gram negative (E.coli)and gram positive (Staphylococcus Capitis) bacteria in the sample. (A) and (B) Two different species of bacteria of equal concentration were mixed in the same sample and O.D value of the solution was measured (n=3). After 17hr of incubation, the filter paper was removed and placed outside to observe the PB color formation on the filter paper. Sample containing the mixture of both bacteria with and without exposing to lysis buffer showed similar O.D values.*

Antibiotic susceptibility testing using ampicillin at 720nm

As an extension to Fig.8, OD values were also measured at 720nm for the antibiotic susceptibility testing that was performed using ampicillin on *E.coli (n=3)*. As expected similar trend of OD values were seen with increase in antibiotic concentration.

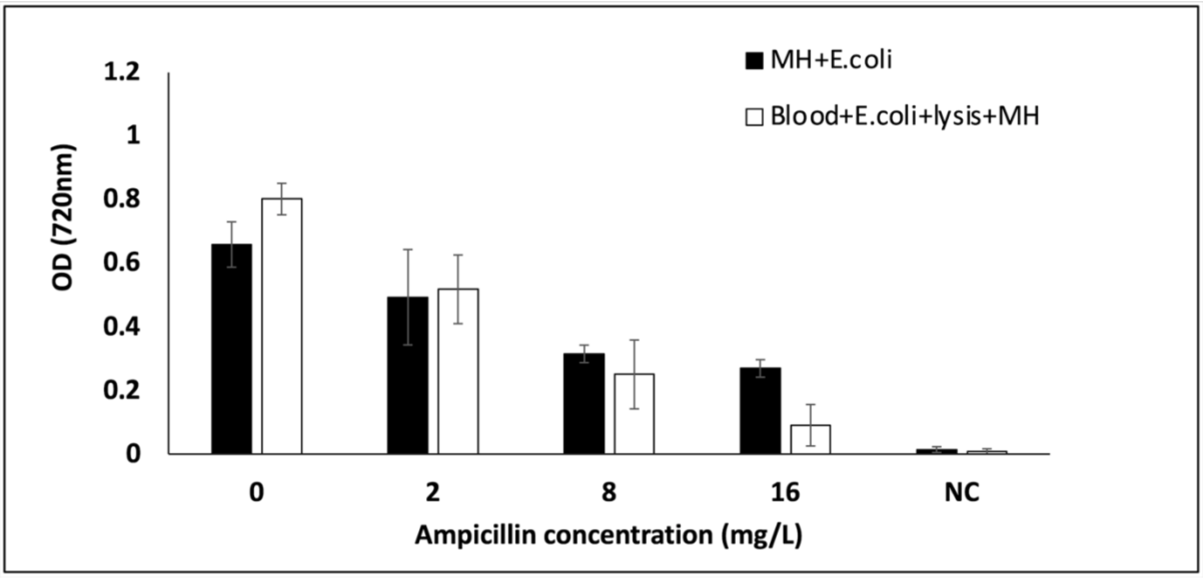

Fig.S6. *Antibiotic susceptibility testing using ampicillin at 720nm. Decrease in OD values were observed as the bacterial growth get suppressed with increasing antibiotic concentration (n=3).*

Antibiotic susceptibility testing using ampicillin for low concentration of *E.coli*

To determine the MIC of ampicillin for low concentration of *E.coli* (1000 CFU/mL), the antibiotic susceptibly testing was repeated and OD values were measures at 600nm and 720nm for 'Blood +*E.coli* + Lysis' as sample (n=3). 'MH + Lysis' was used as a negative control (NC). OD measurements and the PB color formation on the filter paper showed that the MIC was at 16mg/L, showing the applicability of this method to low concertation of bacteria as shown in Fig.S7.

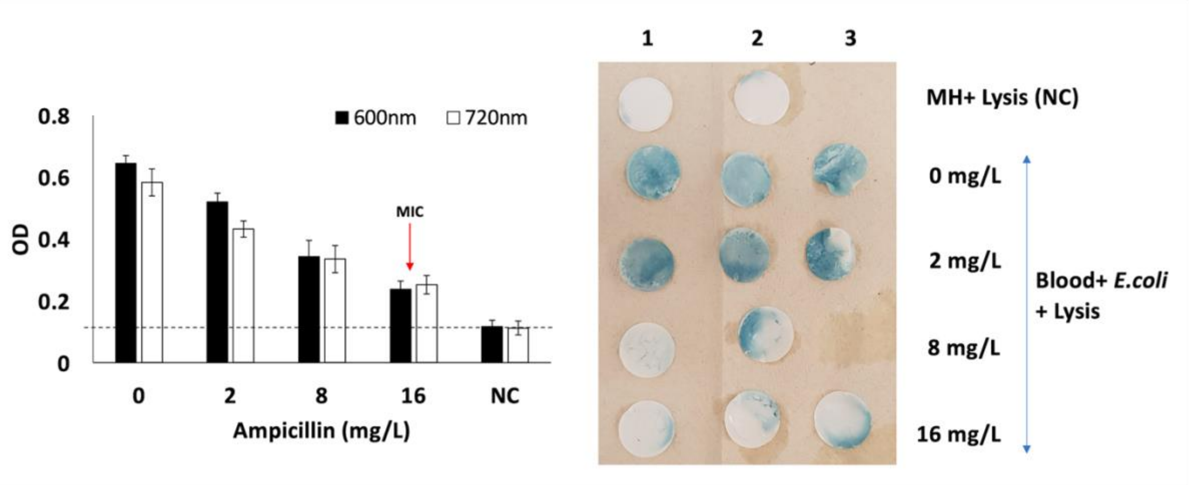

Fig.S7. *Antibiotic susceptibility testing using ampicillin at 600 and 720nm for low concertation of E.coli. Low concentration of E.coli (1000 CFU/mL) was spiked into whole blood and MIC was determined by performing antibiotic susceptibility testing by following the protocol in Fig.1 (n=3). Decrease in OD values were observed as the bacterial growth get suppressed with increasing antibiotic concentration and the MIC was determined to be 16mg/L..*

Antibiotic susceptibility testing using gentamicin at 720nm

Antibiotic susceptibly testing was performed at three different concentration of gentamicin (0.5, 2 and 8mg/L) to determine the MIC of gentamicin for *E.coli*. A similar trend of decreasing OD values measured at 600nm were seen with increase in the concentration of gentamicin concentration as shown in Fig.S6 (n=3). Correspondingly decreased blue color intensity was observed in filter paper with increasing concentration of gentamicin.

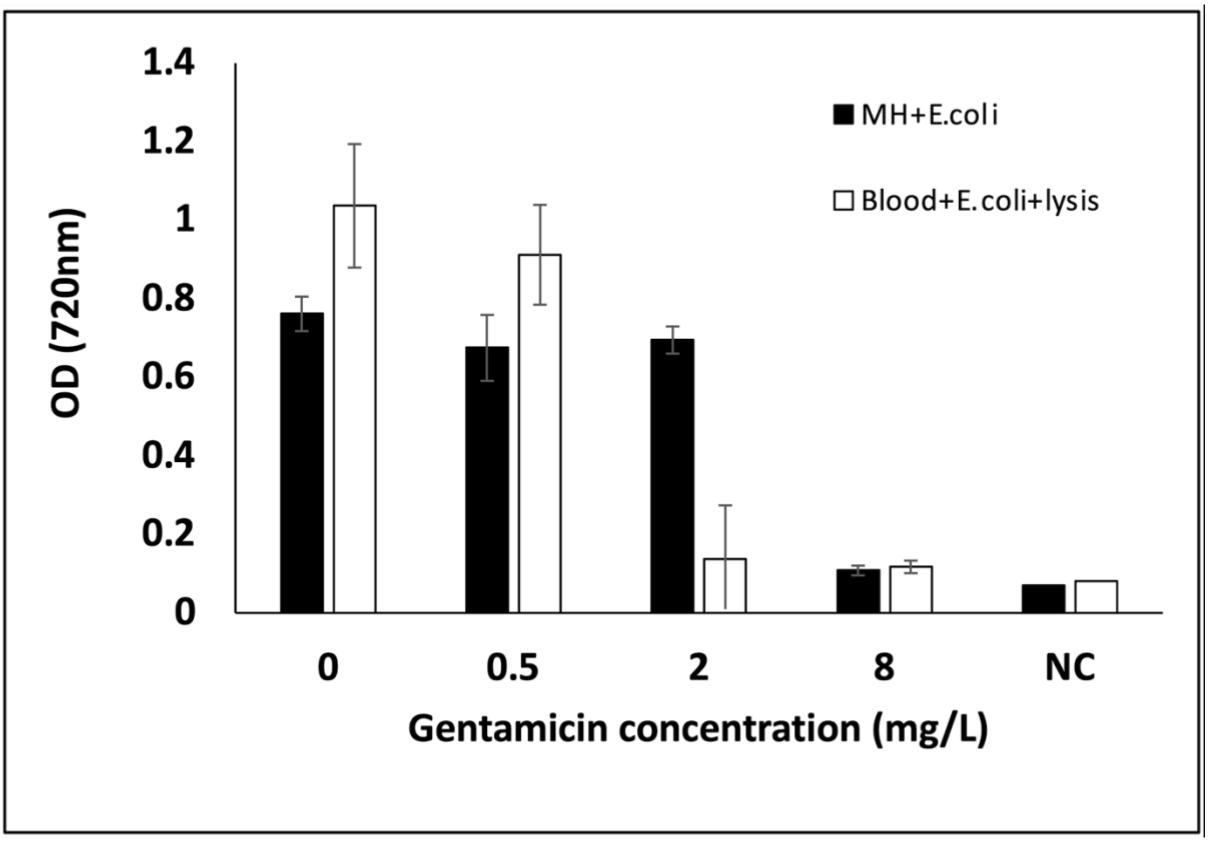

Fig.S8. *Antibiotic susceptibility using gentamicin. It was observed that OD value decreased with increase in concentration of gentamicin. The intensity of PB color on the filter paper also reduced at higher concentration of gentamicin (n=3).*
